# Volatile Organic Compounds in Biological Matrices as a Sensitive Weapon in Cancer Diagnosis

**DOI:** 10.3390/ph18050638

**Published:** 2025-04-27

**Authors:** Arya Ghosh, Varnita Karmakar, Anroop B. Nair, Shery Jacob, Pottathil Shinu, Bandar Aldhubiab, Rashed M. Almuqbil, Bapi Gorain

**Affiliations:** 1 Department of Pharmaceutical Sciences and Technology, Birla Institute of Technology, Mesra, Ranchi 835215, Jharkhand, India; aryaghosh.pharma@gmail.com (A.G.); varnita702133.vk@gmail.com (V.K.); 2 Department of Pharmaceutical Sciences, College of Clinical Pharmacy, King Faisal University, Al-Ahsa 31982, Saudi Arabia; baldhubiab@kfu.edu.sa (B.A.); ralmuqbil@kfu.edu.sa (R.M.A.); 3 Department of Pharmaceutical Sciences, College of Pharmacy, Gulf Medical University, Ajman 4184, United Arab Emirates; sheryjacob6876@gmail.com; 4 Department of Biomedical Sciences, College of Clinical Pharmacy, King Faisal University, Al-Ahsa 31982, Saudi Arabia; spottathail@kfu.edu.sa

**Keywords:** cancer diagnosis, volatile organic compounds, olfactory neural framework, odorant detection, neuronal receptors, insect sensory tools

## Abstract

Diagnosis and intervention at the earliest stages of cancer are imperative for maximizing patient recovery outcomes and substantially increasing survival rates and quality of life. Recently, to facilitate cancer diagnosis, volatile organic compounds (VOCs) have shown potential with unique characteristics as cancer biomarkers. Various insects with sophisticated sensitivities of odor can be quickly and readily trained to recognize such VOCs using olfactory-linked skills. Furthermore, the approach to analyzing VOCs can be made using electronic noses, commonly referred to as e-noses. Using analytical instruments like GC-MS, LC-MS/MS, etc., chemical blends are separated into their constituent parts. The significance of odorant receptors in triggering neural responses to ambient compounds has received great attention in the last twenty years, particularly in the investigation of insect olfaction. Sensilla, a sophisticated olfactory neural framework, is regulated by a neuronal receptor composed of neuronal, non-neuronal, extracellular lymphatic fluid with an effectively generated shell. This review provides an in-depth exploration of the structural, functional, and signaling mechanisms underlying odorant sensitivities and chemical odor detection in the excretory products of cancer patients, addressing current challenges in VOC-based cancer diagnostics and innovative strategies for advancement while also envisioning the transformative role of artificial olfactory systems in the future of cancer detection. Furthermore, the article emphasizes recent preclinical and clinical advancements in VOC applications, highlighting their potential to redefine early diagnostic approaches in oncology.

## 1. Introduction

Cancer is the most prevalent cause of mortality around the globe, accounting for 10 million deaths out of more than 19 million diagnoses in 2020 [1]. According to projections, the number of cancer-related fatalities will surpass 13.1 million in 2030 [2]. Cancerous cells have unique characteristics associated with unbalanced, indefatigable intracellular metabolism, the capacity to constantly nourish themselves by impulses that encourage cell proliferation, or by availing advantage of factors that stimulate the emergence of malignancies [3,4]. At the same time, a distinctive pattern of volatile organic compounds (VOCs) produced by cancer cells owing to their altered metabolic processes serves as a biomarker frame in the diagnosis of carcinoma [5,6]. VOCs are a broad set of carbon-containing chemicals, which are categorized by their boiling point (50–260 °C) and retention period. Apart from the presence of exhaled air, VOCs are also found in biological fluids such as perspiration, saliva, urine, stool, and plasma [7,8,9,10,11]. The sooner cancer is discovered, the greater the probability of a patient recovering from it. The primary aspects of preventing cancer progression involve successful screening along with prompt diagnosis [12,13,14,15].

Furthermore, cancer diagnosis frequently necessitates several sets of evaluations, including those that deal with invasive surgical operations. Alternatively, there are frequent drawbacks to current non-invasive techniques. The ingestion of radiation, a high proportion of positive results are found false, and the potential for over-diagnostics strategies are all risks associated with novel non-invasive methods. For example, screening for lung cancer involves helical computed tomography, which has been demonstrated to pinpoint cancer that can be surgically treatable [16]. Upholding cancer detection techniques represents a potential option to boost outcomes. The likelihood of complete recuperation for survivors is enhanced with the urgency of tumor detection. Few people benefit from accessible screening approaches, since current early detection tools (e.g., colposcopy, magnetic resonance imaging) are frequently invasive or expensive. This emphasizes the critical importance of exploring novel approaches to facilitate the rapid detection of malignancy. Analysis of VOCs represents one of the future-oriented metabolomics methods amongst all omics approaches [17,18,19], offering the prospect of being used as a secure, non-invasive, and precise diagnostic towards the rapid identification of numerous cancers [20].

**Figure 1:**
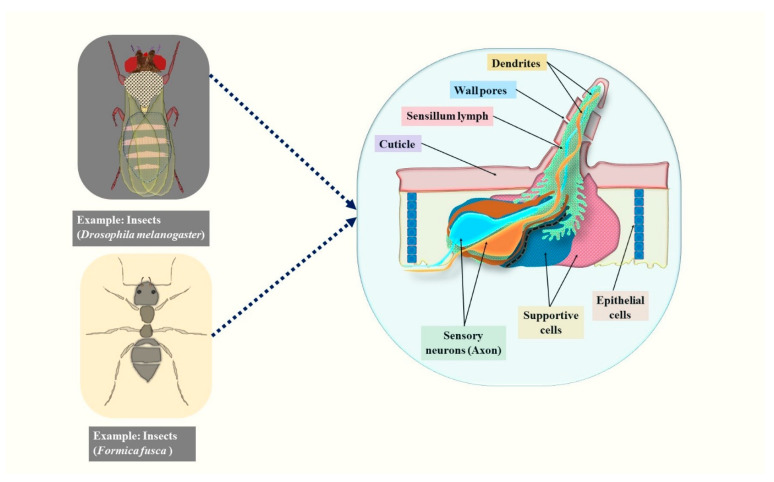
The pictorial representation of the inner structure of insects’ olfactory sensillum.

Animals possess exceptionally sophisticated olfactory mechanisms, capable of distinguishing between intricate odorous substances and recognizing minuscule odorous amounts, which result from millions of years of evolution. Alternatively, insects also possess the potential to distinguish odorous substances because of their very advanced sense of smell; therefore, these are considered less expensive and simpler to raise in under-regulated situations [21,22]. Insects possess highly developed olfactory or sensory networks, which particularly facilitate detection and consequent reactions to volatile stimuli in the atmosphere and play a significant role in their capacity to adapt biologically [23]. Recognizing or biologically defining receptors for olfactory stimuli, the neuronal networks through which they generate, including the regulation of sensory/smell-determine- motions, particularly in *Drosophila melanogaster* (*D. melanogaster*), are all topics that have received an enormous amount of attention over the last 20 years [24,25,26,27,28]. A significant proportion of olfactory stimuli nerve display a pair of independent odorant receptors as well as ionotropic receptors [29,30,31] that comprise odorant-gated ion channels, a phenomenon that is hypothetically enough capable of converting the emission of chemical smells towards depolarization of membranes inside of cells and they function inside intricate sensory cells known as sensilla [32] (Figure 1). As insects are capable of spotting VOCs from lineages of cancerous cells, such insects may be employed as cancer bio-detectors since chemical smells from various cancerous cell lines cause distinct sensory receptor responses in the signal [33]. Similarly, carpenter ants are exceptionally skilled in distinguishing distinct odorants and additionally differing quantities of the same chemicals [34,35,36]. As evident from the current body of literature, no comprehensive review has yet explored the application of insect olfactory sensing systems in detecting cancer-associated VOCs. The present review addresses this gap by highlighting bioinspired olfactory strategies and their emerging relevance in oncological diagnostics.

This review highlights key aspects of insect traits, including their morphology, olfactory biochemistry, and neurobiological properties of olfactory odorant stimuli to learning, recall, and obliteration, including chemical compound detection as sensory bio-detectors. Also, the review focuses on the structural, functional, and signalling bases of odorant sensitivity and VOC detection in cancer-related excretory products, addressing diagnostic challenges and strategies to overcome through advanced technologies. Additionally, it highlights the role of artificial olfactory systems in the future of cancer detection and clinical advances in VOC applications, underscoring their potential to transform early cancer diagnostics.

## 2. Volatile Organic Compound and Available Detection Techniques

An investigation into diagnosing cancer at its early stages has been initiated over a period of 20 years. Therefore, depending on the theory, certain VOCs capable of being traceable by biochemical analytical methods may be produced when a malignancy grows via several factors, such as modified metabolic processes in cancerous cells [37]. In order to understand the physiological and pathological mechanisms, VOCs are currently attracting the attention of researchers, alongside developing methods for analyzing alterations in concentration or generation of VOCs in different physiological matrices for diagnosis and pharmacological surveillance.

Each creature, from microorganisms to animals such as vertebrates, utilizes olfactory sensation [38]. Pheromones represent an instance of a compound that an animal willingly emits. Other compounds can be associated with an individual’s metabolic processes [39]. Alcohol and other organic chemicals must be volatile in order to be detected in expired air [40]. VOCs are not just applied to breath-based detection. Two fundamental analytical methods, gas chromatography with mass spectrometry and liquid chromatography with tandem mass spectrometry (LC-MS/MS), have been implemented. Alternatively, synthetic olfactory mechanisms of fabricating electronic noses are employed to identify these chemical substances. A few of these techniques used in the determination of VOCs in different disease states are summarized in Table 1, Table 2, Table 3, Table 4, Table 5 and Table 6.

While gas chromatography–mass spectrometry (GC-MS) is involved in the VOC analysis, the specimen are initially processed through GC, essentially distinguishing the various components in the solution, before being subjected to MS analysis to determine their identities. In due course, the chemicals travel via a capillary column with the aid of an inert gas as the operational temperature of the chamber containing the column is elevated. Lesser volatile substances require longer to complete the process than higher volatility substances. According to the nature of the column, certain molecules can come into contact with it, which disrupts its motion in a unique manner [63,64]. Consequently, chemical blends are dispersed into their individual components through gas chromatography. Ionization serves to break down the compounds, and their mass and charge are determined by passing them through an electrical field to the MS. Each chemical ends up in a distinct and distinctive splitting structure. Similarly, the chromatographic system of liquid chromatography–mass spectrometry/mass spectrometry helps in separating the compounds. In contrast, the mass spectrometry/mass spectrometry system helps identify the VOCs of interest generated or altered during the diseased state.

In this context, to detect the initial stages of lung carcinoma, a volatolomic study was conducted using liquid chromatography–mass spectrometry/mass spectrometry method. A stochastic analytical estimated model and characterization of target-specific issues were used to evaluate the discriminating precision of detected VOCs. In this study, the breathed VOCs from earlier lung carcinoma patients were compared with healthy participants prior to surgery, 3–7 days following surgery, 4–6 weeks later while fasting, and 1 h after a meal to determine how the recommended method performed in contrast with current strategies. Invasive adenocarcinoma of the pulmonary was identified to have the greatest variability in concentrations of seven VOCs, including 3-hydroxy-2-butanone, glycolaldehyde, 2-pentanone, acrolein, nonaldehyde, decanal, and crotonaldehyde, only 1 h after a meal [41]. A couple of VOCs, propan-2-one, which was raised, and carvone, which was dropped, were identified to undergo significant changes utilizing both alkaline and acidic techniques [42].

Nowadays, electronic noses, often known as e-nose, represent an additional significant method of VOC testing. Numerous detectors compose each one, and they all respond differently to the component that is being shown. The concept is identical whether polymer-based or metal-based detectors are applied. VOCs adhere to the detectors that correspond to their affinity for them when they are examined using an e-nose. Following this association, the detectors’ electrical conductibility and resistivity vary, developing particular electrochemical rhythms. A recent study on the detection of VOCs in urine samples that could potentially be used to differentiate female cervical carcinoma from healthy females through an e-nose and to detect possible biomarkers by GC-MS was conducted. In this cross-sectional study, a sampling of 12 healthy females, 5 females with cervical intraepithelial neoplasia, and 12 with having cervical carcinoma was performed. According to the GC-MS study, 33 compounds belong to the class of cervical carcinoma, while a few of these substances have been linked to other carcinomas. Overall, utilizing GC-MS analysis and the e-nose technique, this investigation lays the groundwork for the creation of a cervical carcinoma detection tool that is readily available, non-invasive, accurate, and precise [62]. However, in order to carry out the evaluation, predictive modeling, artificially created neural networks, and numerous variable methods must all be integrated. The incorporation of animals in the determination of VOCs has brought a new technology that offers several advantages over the existing methods.

## 3. Olfactory Network Assessment for Odorant Detection in Animals

Living organisms frequently employ detecting smells; thus, hunting, copulation, or finding mates are executed easily. Although they have diverse developmental tracks, vertebrates and invertebrates both have sensory receptor networks [65,66]. Sensory appendages, including the airway passage in vertebrate animals and the feeler in invertebrates, detect odorous chemicals in the peripheral region [67]. Odorant chemicals, including VOCs, are sensed at the outermost layer of specific structures through neuronal sensory receptors, which trigger action potentials that transfer the sensory data to an individual’s neurological system. The message then travels across the junctions between peripheral and central neuronal cells prior to reaching the higher cerebral centers. The primary olfactory center of insects, the antennal lobe, is formed with several spheroidal neuropilar units. This junction is called a glomeruli, which connects the peripheral receptor neurons to the interneurons from the antennal lobe [68]. Glomeruli are found in the antennal lobe of creatures and the olfactory bulb of mammals. Odorants activate particular neuronal pathways, where they are subsequently transported to greater processing areas identified as mushroom-like structures and lateral spikes in invertebrates and amygdala in mammals. The neuronal sensory receptors, especially those employed for olfactory recognition, are specialized. These neuronal sensory receptors in insects connect to the antennal lobes of the macro-glomerular network. A cognitive reaction is generated by a trigger based on the perception of the individual signal and previous exposure to it. Even though an odorant is absent from or encounters no physiological significance in an animal’s native habitat, it is nevertheless feasible for that species to acquire the ability to respond and detect any given odor via adaptation [69]. In this manner, the insects are able to detect specific smells with the help of specific sensory receptors.

### Animals as Bio-Detectors

Creating a non-invasive, affordable, and effective method for the detection of early-stage cancer poses a key challenge with vital healthcare concerns. A distinctive odor of VOCs formed by malignant cells due to their metabolic modification may be exploited to establish a cancer diagnostic biomarker [61]. Various animals, including canines, rodents, and certain insects, demonstrate exceptional olfactory sensitivity to volatile organic compounds (VOCs) emitted by cancerous cells. Numerous published cases prove that dogs with a well-developed sense of smell can diagnose cancer by detecting VOCs released by cancer cells. Using cancer-specific VOCs, the accuracy of the dog in differentiating between a healthy and diseased person is quite impressive. Despite this, their capacity to identify cancers can be valuable, particularly when there is an absence of or limited access to early diagnosis instruments or in types of cancer that do not have efficient early identification procedures [70]. In one such study, sniffer dogs have shown that they are useful tools in an early non-invasive diagnosis of lung cancer and distinguished cancer-specific VOCs in breath and urine. As such, this capability presents itself as a potential additional modality to lung cancer screening, where normal diagnostic methods may not be possible or available for patients [71]. Small mammals such as rodents are preferred for lab-based cancer detection research because of their high olfactory receptors (ORs) and ability to undergo olfactory learning; they can be trained to correctly detect cancer odor. An animal nose sensor system using trained rats demonstrated high accuracy (82%) in detecting toluene, a lung cancer odor indicator, with consistent performance across varied environmental conditions and long-term memory retention [72]. Since insects may procreate quickly, inexpensively, and with ease, they were explored as the laboratory biodetectors for the aforementioned method. Fruit fly species were employed to examine this challenge [33]. Researchers have demonstrated that a fruit fly’s olfactory system can detect and differentiate cancer cells from healthy ones through direct, quantifiable calcium imaging of antenna responses, offering a sensitive, objective alternative to animal-based cancer detection [73]. With the aid of in vivo calcium image processing, the investigators had the opportunity to demonstrate that when subjects were constrained and exposed to the smells of cancerous cells, they formed unique neurological structures that were significantly distinct from those produced by others when they were exposed to healthy controls [74]. Also, honeybee olfactory neurons were successfully used to classify human lung cancer biomarkers at low concentrations, achieving high accuracy in detecting non-small cell and small cell lung cancer cell lines [75]. Thus, owing to the inexplicable highly specialized olfactory system in insects, it becomes essential to explore the sensory mechanisms of the insects, which are summarized in the connecting section.

## 4. Insect Olfactory Neurobiology: Molecular Insights into Pheromone Detection

Olfactory sensory receptors initiate odorant-induced neural activity by triggering ligand-dependent membrane depolarization across various cell types. In natural settings, olfactory signalling is shaped by the combined presence of multiple odor molecules and chemically diverse compounds, along with the dynamic regulation of receptor sensitivity. The sensilla lymphatic fluid contains ionic amalgamation (fluid-filled), rich in released amino acids along with proteoglycans, where odors need to pass during penetration to sensillum [76]. The physicochemical properties of odorant molecules determine the rate at which ions may penetrate the sensillum, pass via lymph, and be integrated into the olfactory sensory neuron (OSN) layer [77,78]. Olfactory-binding proteins (OBPS) recognize and bind a structurally diverse range of odorant molecules, each with distinct binding affinities determined by their chemical characteristics. Ligand binding induces specific conformational alterations in OBPs, which facilitate odorant transport and contribute to the initial steps of olfactory signal transduction [79,80]. The OSN exists when they discharge odor to the receptors, potentially provoked by regional pH variations close to the cilia membranes or through hypothetical interactions of OBPs with cilia proteins found in the membrane. This is how OBPs have historically been thought to function: they communicate with and convey hydrophobic ligands through the lymph fluid to the OSN [80]. Electrophysiological and psychological responses to the pheromone cis-vaccenyl acetate (cVA) in *D. melanogaster* depend on the OBP LUSH (also known as OBP76a). While LUSH could assist in the transport of cVA to the homologous receptor (OR67d), it is not a crucial component of the signal transduction system because supraphysiological amounts of pheromone remain capable of triggering some neuronal activation [81,82].

**Figure 2:**
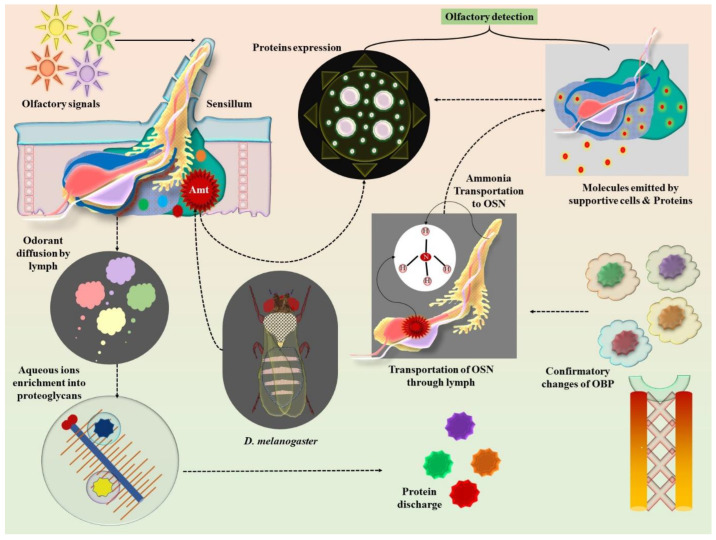
Schematic representation of olfactory sensillum response on odorant detection. Odorant molecules are integrated into the olfactory sensillum, which is dispersed by sensory lymph. The harmonizing alternation of OBP due to its molecules bound to their surface as a result of enrichment of aqueous ionic collage to proteoglycans and release of proteins. The hydrophobic molecules bound OBP to carry out OSN through lymph, which permits the signals (odors) to be delivered to the receptor, supplying signaling molecules through proteins as well as supportive cells. The coeloconic sensillum (Supportive cells), also called ammonia-sensing neurons, is where the protein molecule Amt in *D. melanogaster* is addressed. Amt plays a role as a key membrane conveyor to take out the ammonia from lymph to dendrites at a low basal concentration for olfactory detection. Misplacing/dropping Amt triggers the ammonia response.

Analysis of family members expressed across multiple sensillar types in *D. melanogaster* has shown nuanced and occasionally unanticipated functions in comparison to pheromone-associating OBPs. Greater biological responses to odorants showed up in one basiconic sensillum group (ab8) when OBP28a disappeared, indicating a potential function in regaining the ability to regulate odorant-induced activation [83]. A certain percentage of OSNs experienced a subsequent inactivation of neurological responses after odor elimination as an outcome of concurrent deletion of both expressed OBP83a and OBP83b; notably, this characteristic was restored via restate of each protein independently [83,84]. Supportive cell membrane proteins may aid in sensory signals in combination with the chemicals released by these cells. The coeloconic sensillum group of supportive cells present in *D. melanogaster* contain an ammonia-sensory neuron, which is assumed to be the cells in which the ammonia transporters (Amts) are expressed. According to a genomic study, Amt depletion results in significantly reduced responses to ammonia stimuli. A possible explanation would be that Amt eliminates ammonia from the lymphatics to minimize the baseline levels of this molecule adjacent to the OSN dendrites, hence reducing the static adaptability of ammonia-sensory nerve cells (Figure 2) [85]. This mechanism underscores the role of ammonia clearance in maintaining olfactory sensitivity, which is critical for precise pheromone detection. Such fine-tuned detection systems may inspire biomimetic approaches for sensing volatile cancer biomarkers. The most commonly encountered member of the cluster of differentiation 36 (CD36) group of proteins found in mammals is sensory neuron membrane protein 1 (SNMP1), a two-pass membrane-based protein molecule. In accordance with its manifestation and ciliary adaptation in pheromone-sensory neuronal cells, two features of SNMP1 are widely preserved in insects that initially emerged in moths [86,87,88,89,90,91]. The OR67d intermediated effects on cVA were shown to require SNMP1 by metamorphosis research in *D. melanogaster* [88,92]. The fact that highly concentrated amounts of this pheromone can circumvent the need for SNMP1, as with the OBP LUSH, indicates that it is not an absolutely necessary component of the receptor unit. Other insects also possess the potential to retain SNMP1’s crucial function in pheromone recognition, as it does in *D. melanogaster* [89,91].

Mechanical investigations into SNMP1 activity have been driven based on the fractional ability of mouse CD36 homolog to restore Snmp1 variants within *D. melanogaster* [90]. The SNMP1 ectodomain is anticipated to have a hydrophobic space by a homologous system using a crystal frame of the CD36 protein molecule lysosomal integral membrane protein (LIMP2). This cavity may serve as a pathway for the transportation of hydrophobic olfactory chemicals from external lymphatic cells to a tightly organized membrane [88,90,93,94]. Despite the fact that the precise mechanism is still unknown, it is possible that the biochemical obstacles of emphasizing typically broad as well as extremely hydrophobic olfactory ligands at the outermost layer of the OSN walls account for the essential need for OBPs along with SNMP1s in the identification of pheromones. Following receptor triggering, additional messenger molecules possess the ability to keep altering olfactory neuronal activity. It remains unclear exactly how ant inotropic olfactory sensory receptors can transmit signals with sensitivities similar to those of mammal metabotropic chemosensory receptors without using secondary messengers to amplify the signal [95]. A recent report revealed that the Pickpocket 25 (PPK25) degenerin/epithelium Na+ channel increases ligand-induced impulses downstream of specific sensory receptors. The biological response of Or47b OSNs, a subclass of pheromone-sensory neurons implicated in mating actions, is altered in *D. melanogaster* by genetic PPK25 deletion or overextension [96,97,98]. These consequences are apparently calmodulin-based due to pharmacological blocking of calmodulin or mutagenesis of a calmodulin-binding domain in PPK25, which imitates the deletion of PPK25. It is important to note that PPK25 serves a similar role in gustatory sensory neurons (GSNs), which recognize non-volatile chemicals, and OSNs that contain ionotropic receptor 84a (IR84a), which detect food-derived smells that promote courtship activity [98,99]. The molecular mechanisms involving SNMP1-mediated transport of hydrophobic odorants and PPK25-dependent amplification of receptor-evoked signals are fundamental to maintaining the sensitivity and selectivity of insect olfactory systems. These pathways enable the recognition of lipid-derived and volatile pheromonal compounds, some of which possess chemical features analogous to VOCs emitted by cancer cells. Understanding these molecular adaptations in insect olfaction provides a valuable framework for developing biomimetic olfactory sensors for non-invasive cancer detection based on volatile metabolite profiles.

## 5. Olfactory Biochemistry and Impulse Sensitivity of Regulatory Receptors

**Figure 3:**
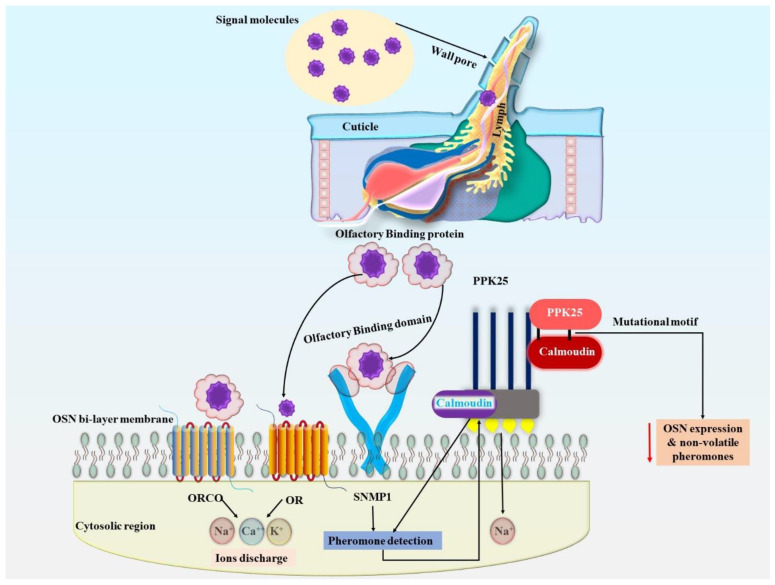
Molecular signalling pathway of olfactory proteins towards the receptors. The representation of the olfactory transduction pathway concerning insect olfactory sensory neurons (OSNs) to better clarify the complexities of the identification of volatile and non-volatile pheromonal molecules. As the pheromonal ligands pass through wall pores and enter the cuticular space, they are bound by the OBPs and transported to the OR complex present on the ciliary membrane. Activated by ligand, the cloned OR-ORCO heterodimer functions as an ionotropic channel to provide the cells rapid access to Na^+^, K^+^, and Ca^2+^ ions, which cause cytosolic depolarization. Hydrophobic pheromones are transported by SNMP1, a transmembrane protein; there is also a PPK25 channel that is regulated by calmodulin and should intensify the signal. Altered PPK25 or the calmodulin-binding domain affects the OSN’s ability to respond, leading to poor sensing of non-volatile, high-affinity pheromones used in fine-tuning insect behaviors.

Two individual biological mechanisms account for the OSN signal. First is the odorant-based sensory receptor activation, then ion movement along with depolarization of cilia surface, followed by progression of generated nerve impulses from the original stimulus towards the OSN axon via voltage-gated channels (Figure 3) [100,101,102]. Variations within the local field potential (LFP) of sensillum may be utilized to figure out the initial stages of these mechanisms. These alterations reflect transitory electric impulses in the sensillum formed by OSNs and even inputs by ion movement of supportive cells [101,102]. The unique connective features between odorant molecules and sensory receptors become apparent in LFP factors, which symbolize signal transduction factors. The unique features of the interactions between smell ligands and receptors are reflected in LFP dynamics, which represent signal transduction qualities. However, a linear filter known to be uniformly distributed across several OSN groups may clarify the periodic pattern of spiking/action potential [101,103]. Spike rate tends to be the only indicator employed for capturing odorant-inspired neurological responses in several nasal cellular investigations, which do not evaluate LFP [101,104,105]. Although spikes indicate a signal that is conveyed inside the neural network in the brain, understanding actions to genuine odorant triggers requires in-depth comprehension of periphery OSN activity. Largely concentrated chemical-containing spaces of air form the airborne plumes that make up smells. Different OSN responses, such as sensitivity with potent stimulus and sensitivity for recurrent mild stimulus, are elicited by such spatially difficult stimulation array [98,100,101,103,106]. In addition to having extremely diverse dynamic transformations, LFP and spike frequency also tend to change according to several features of the olfactory stimuli [101,103]. While the dynamic mechanism of each element’s adapting responses is different, variability associated with odorant stimuli affects LFP and spike frequency [103]. Since LFP and neuronal spikes are closely related events, even though their kinetics are different, variations affecting their frequency exhibit comparable dynamics compared to LFP [104]. Collectively, these evaluations illustrate how intricate OSNs are at encoding various olfactory cues and demonstrate that solely detecting spike rate does not reflect OSN response or the ability of humans to comprehend the process of developing olfactory-induced neurological action. It is still unknown which molecular entities underlie the dynamic pharmacological characteristics of OSNs. Most studies have concentrated on unraveling the actions of ORCO, revealing that sensory modification depends on altering both receptor position and sensation. Despite being studied across a large period, these co-receptors appeared to be diminished from cilia following continuous odorant exposure. Calmodulin can be associated with action-based modulation of ORCO position because its cilia location is affected by calmodulin RNAi along with the transformation of a putative calmodulin-binding domain in ORCO’s subsequent inner loop, resulting in deficits with odorant-induced action [107]. The significance of calcium activation or simply calmodulin within ORCO-based sensitivity of neural cells following recurrent olfactory stimuli and sensory modification, exposing neuronal cells, has been further supported by biological studies [108]. A trio of putative spots for phosphorylation may be further found within the ORCO loop. The signaling potency of ORCO in heterologous cells is decreased by mutagenesis of these spots, and OSN sensitivities and or physiological reactions towards odors in vivo are also impaired [109,110,111]. Furthermore, odorant sensitivity is hindered by mutagenesis, which alters the phosphorylation regions of ORCO. Desensitized OSN triggers the in vivo dephosphorylation amongst the majority of these regions, i.e., S289 [106,109,112]. OSN sensibility can be impaired in vivo due to the mutation of ORCOS289A gene, while the degree of desensitized OSN following odorant exposition is diminished by an ORCOS289D gene mutation [107]. These findings highlight the intricacy of olfactory sensory receptor oversight, which influences time-dependent responses and defines OSNs; however, they additionally emphasize how difficult it is to distinguish impacts on the receptor site and its function. Though N-glycosylation is believed to be associated with the modulation of ionotropic receptor (IR) sites along with activity, it remains primarily unexplored how various forms of odorant receptors are modulated by molecular form [113]. According to electrophysiological analysis, OR and IR neural cells exhibit distinctive time-bound response traits, particularly those which seem to be strongly influenced by receptors [108,114,115]. Furthermore, additional studies need to be conducted regarding the immediate effects of networks associated with sensilla progression, such as lipid flippase ATP8B or Hedgehog signal. Modern studies have revealed the interconnections in the functional aspects of several OSNs inside similar sensillum and independent regulative processes within OSNs. When other nearby neuronal cells become activated, the signalling associated with a single OSN across several sensillar types is suppressed. Unfortunately, there is neither confirmation of junctional space between coupled OSNs nor any mechanism for avoiding the antagonistic linkages among two distinct OSNs when synaptic signaling is obstructed [116]. These findings imply that sensory blocking occurs due to an ephaptic connection, wherein the conductivity inside a single neuron modifies the local electrical field in a way that inhibits depolarization of adjacent neurons [116,117]. The concurrent monitoring of both sensilla linked by a metal electrode indicates that persistent installation of a neuronal cell within a single sensillum might be obstructed by stimulation within the neighbouring sensillum, providing evidence in favor of this concept. Furthermore, the amalgamation of this evidence using CryoChem’s EM evaluation of certain neuronal classes demonstrates that the blockade is greater once it propagates from greater OSN to lesser OSN [117]. Greater neurons are anticipated to possess lesser signaling resistance as well as a larger terminal region in order to enable greater peak LFP, suggesting a formerly unrecognized connection between OSN morphological and physiological properties. The justification of a disproportionate link between the two seems a viable one. The implications associated with ephaptic coupling on odorant coding, especially for intricate naturalistic odorant combinations, will be investigated in greater depth in future research. Research on GSNs found in the Bombus terrestris (bumblebee) offers intriguing novel perspectives into the mechanisms underlying communication amongst neural cells in the identical sensillum. When large quantities of sugar become the stimuli, observations obtained from extremely charged sugar-detecting nerve cells inside the mouthparts’ type A sensilla display a unique exploding structure for spikes [118]. An individual spike of another neuron within this sensillum indicates the closure of each spike outburst. Conversely, against the emphatic suppression outlined in sensory sensilla, the addition of a junctional space blocker inside the sensillum causes the primary neuron to activate continuously in response to sucrose as well, indicating that the secondary neuron ceases the neuronal firings of primary neuron through synapses that conduct electrical impulses. The capability of insects (e.g., bees) to maintain their feeding behavior on sugar–honey could be explained by the observation that this burst sequence of discharge inhibits neural desensitization [119]. Alternatively, ants have also been employed in the detection process of cancer biomarkers by the use of their sensory receptors [120].

## 6. Olfactory Morphology of Ants and Its Potential Applications

Since ants have access to such a wide variety of sensory receptors (odorant), they employ a sense of smell in essentially every situation [66]. Olfaction plays a role in breeding, hunting, alerting, and defending against challengers, as well as individual patrolling and separate identification of associated players [121,122,123,124]. Ants can modify the process to detect and acquire information about their companions by employing pheromones [125,126]. A couple of species were investigated for their capacity for olfactory sensory perception in spite of the vital relevance of the sense of smell for ants.

### Exploiting Insect Neurobiology for Early Cancer Sensing

Studies of ants in the detection of cancer have crossed the boundaries of laboratories. A recent study indicated its use in the detection of cancer in clinical samples. In due course, the ants underwent three training cycles in an annular space where the chemical odorant of an ovarian cancerous cell specimen (IGROV-1) was established in Dulbecco’s modified Eagle medium (DMEM), which was linked to an incentive of sugar mixture. The researchers observed that the ants were occupied by examining two distinct odors, the odor of the resistant strain (RS) of the cells in question (IGROV-1), while the odor of the culture media (DMEM) (fresh odor) was tracked by the investigator in a similarly shaped annular space. Additional controls consisted of a pair of vacant tubes. Ants showed signs of cell recognition throughout these recall tests by spending a disproportionate amount of period close to the stimulus odor (cancerous cells) compared to the culture media only [61].

**Figure 4:**
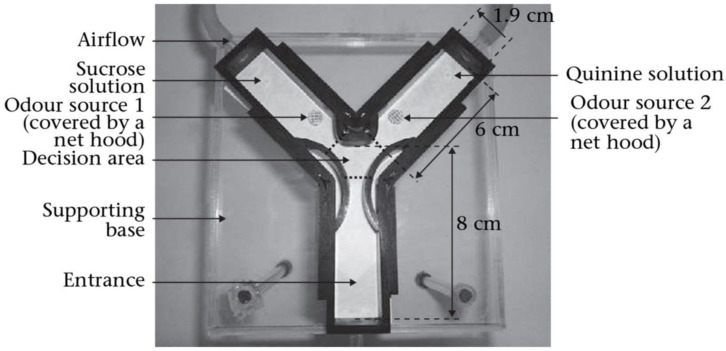
Top view of the acrylic Y-maze used for conditioning ants in an olfactory discrimination task. Each ant was transported to the entrance zone of the maze, where it was released. The ant moved towards the decision area, delimited by the dashed lines on the figure, where it had to choose between the two odors. The airflow ensured odor diffusion. Odor detection at the decision area and/or arm entrance was followed by the reinforcement assigned to each odor (sugar solution or quinine solution). Owing to the spatial arrangement of odor and reinforcement, ants therefore experienced first the odor and then the reinforcement (forward pairing) [131].

*Camponotus aethiops* (*C. aethiops*) served as the experimental subject for the initial tethered Ant technique [127]. Ants were trained to link a positive affirmed smell (RS+) with a mixture of sugar (US+) and a negative affirmed smell (RS−) with quinine (US−) through divergent training [74,128]. A series of 12 training experiments ((6 RS+) and (6 RS−)) revealed outstanding capability to learn in *Camponotus fellah* (*C. fellah*), as evidenced by their ability to keep a learned connection for a period of 72 h [129]. Protein formation inhibitors were used to demonstrate that the recall created after training represented true long-lasting recall since true long-lasting recall requires de novo protein formation [130]. By using free movement theories, the ants are set up in an atmosphere that seems more analogous to their natural environment. *Camponotus mus* and *C. fellah’s* sensory (olfactory) memory were examined using a distinct Y-maze setting (Figure 4) [131]. Ants had no trouble distinguishing between positively affirmed smell and the opposite group following a set of 24 training studies, which took place moments after the conditioned process was completed. Afterwards, *C. fellah* revealed and created memories that remained for a maximum of 72 h following adaptation with a similar model [132]. Round courts are additionally beneficial in studying free-movement frameworks. Throughout the recall assessments, *C. aethiops* liked to indulge in a longer period close to cuticular compounds (hydrocarbon) spectra used as RS instead of an unfamiliar odor [133,134]. In terms of specific sensory progress, *Camponotus* ants have received the greatest attention. The nest-mates (labours) in this species are fairly big, and they initially demonstrated an excellent learning skill [74,128].

## 7. Challenges and Advanced Strategies to Overcome Cancer Diagnosis via the Detection of Volatile Organic Compounds from Biological Matrices

Detecting VOCs from biological matrices such as blood, urine, or breath presents several challenges due to the complexity and variability of these biological systems.

### 7.1. Complexity of Biological Matrices

Biological fluids like blood, urine, and breath are complex matrices of thousands of endogenous and exogenous compounds that might interfere with the identification of cancer-specific VOCs [135]. Such matrices are complex, making it challenging to differentiate between normal metabolic byproducts and disease-specific biomarkers; thus, highly selective and sensitive detection methods are needed to be able to differentiate between the slight differences in VOCs [136].

One of the most studied applications of VOC detection is lung cancer diagnosis through exhaled breath analysis. However, exhaled breath contains hundreds of VOCs, many of which are not cancer-specific and can result from diet, environmental exposure, such as air pollution or smoking, or other non-cancerous conditions, such as infections and chronic obstructive pulmonary disease (COPD). This overlap complicates the identification of cancer-specific VOCs [137]. Several researchers studied VOCs in the breath of lung cancer patients using gas chromatography–mass spectrometry. The work by Tsou et al. demonstrated some elevated VOCs in lung cancer patients, which are alkanes and benzene derivatives. All such compounds were found to be present in patients with non-malignant lung diseases. The study requested more focused strategies and a greater number of patients to differentiate between lung cancer and other diseases by VOC signature [138]. In this regard, scientists began to use machine learning with pattern recognition algorithms by analyzing intricate VOC patterns rather than using the single VOCs themselves [46]. It directly enhanced the specificity of lung cancer detection by identifying distinctive VOC patterns associated with the disease. For example, Dragonieri et al. (2009) built up an e-nose that was utilized to differentiate patients with lung cancer from healthy controls in terms of complex breath VOC patterns [139]. While not perfect, this technique showed promise in enhancing the specificity of breath-based cancer diagnosis despite the complex background of non-cancer VOCs.

### 7.2. Diminished Concentration of Volatile Organic Compounds

The very low concentrations of VOC typically demand highly sensitive analytical methodologies, such as GC-MS or advanced e-nose sensors, for accurate detection, which is the major challenge in this field. Biological matrices also encompass a large number of interfering compounds whose signals may interfere with pertinent VOC signals, thus preventing the identification of the cancer-specific VOC in the presence of other VOCs [135].

Blood is one of the most challenging biofluids to analyze due to the presence of these proteins, lipids, and other metabolites, which could interfere with VOCs associated with cancer. In ovarian cancer, such VOCs present in the blood are at a very low concentration, making their identification difficult. Moreover, blood comprises volatile compounds from distinct organs and metabolic procedures, and as a result, identification of certain VOCs that have a direct link with ovarian cancer is complicated [140]. Raspagliesi et al. employed head-space solid-phase microextraction in combination with GC-MS to analyze blood samples of ovarian cancer patients. Several VOC biomarkers were identified, including fatty acids and short-chain aldehydes, and they were even found in the blood of healthy individuals. The highly complex composition of the blood matrix, compounded by the relatively low concentration level of potential cancer-associated VOCs, posed significant underlying analytical difficulties in establishing a precise diagnostic test [141]. To identify the compound profiles of the samples, the researchers used two-dimensional gas chromatography (2D-GC). This technique enhanced VOCs’ selectivity over the complicated blood sample matrix with enhanced sensitivity [142]. This particular technique enabled the differentiation of even slight changes in VOC among patients and healthy controls, thus leading to enhanced accuracy in identifying ovarian cancer.

### 7.3. Benign Interference

Another important consideration resulted from the sampling and sample preparation steps in the standardization process. VOC profiles are extremely dependent on environmental conditions, handling practices, and even patient-related factors such as diet and the patient’s medications; they are not highly reproducible. Consequently, the studies must adhere to strict and replicable sampling methods to allow for comparison and biomarker identification [143].

Colorectal cancer (CRC) using VOCs from exhaled breath is another area of research and the big challenge here is the complexity of the matrix. Based on the observations made by the present work, specificity has emerged as the major characteristic of VOCs produced by the gastrointestinal system due to the influences of gut microbiota, diet, and inflammation. Some of the other gastrointestinal disorders (GITs), such as inflammatory bowel syndrome (IBS) and inflammatory bowel disease (IBD), might possess VOC profiles similar to what has been identified as potential biomarkers for CRC, and thus the already enhanced specificity of the diagnostic becomes even harder to achieve [144,145]. In the quantitative study of the breath of CRC-diagnosed patients, Wang et al. further discussed and compared the breath of the healthy counterparts and patients with non-cancerous gastrointestinal disorders in 2013. Among these VOCs, the researchers found aldehydes and hydrocarbons linked to colorectal cancer. However, it was also observed that many of the VOCs with increased levels of benign gastrointestinal disorders, particularly benign gastrointestinal diseases, entail a high rate of false-positive results [146]. To enhance the ability to diagnose diseases, the researchers added VOC with other biomarkers, including fecal occult blood tests and genetic markers, hence a compound biomarker. It improved the distinction of CRC biomarkers, their sensitivity and specificity, and eliminated the interference of benign diseases in VOC assessments [147]. It showed that the optimum approach was combining VOC analysis with other analytical tools to increase accuracy in multi-component biological samples.

### 7.4. Instability Concerns

Moreover, the volatility of some of the VOCs, or the fact that they are prone to decomposing in storage or when in transport, also poses a challenge in their detection. Adsorption and accumulation on the inner surfaces of the containers also make it difficult to obtain some necessary diagnostic data for certain VOCs. These aspects must be considered while simultaneously identifying new material types that can still protect the integrity of VOCs [6].

The nature of Lung cancer detection by exhaled breath analysis is very sensitive to the stability of VOCs. Since most VOCs are highly reactive compounds, they decompose or escape into the atmosphere before testing whether samples are not collected, handled, or stored optimally. Temperature, humidity, and the collection container were shown to have a major impact on VOC stability in breath samples [148]. In the scientific work of Keogh et al., the authors attempted to evaluate the possibility of using VOCs already present in exhaled air to detect lung cancer; the authors of the work estimated the stability of VOCs in exhaled breath. As stated, some of the specific VOCs associated with cancers such as alkanes and aldehydes are short-lived and are likely to disappear within a few hours of sample collection. The rate of degradation that occurred depended on the storage: there was greater loss of VOCs when stored at room temperature compared to lower temperatures such as −80 °C. These led to some unpredictable results; therefore, the breath VOCs are not very reliable for cancer diagnosis [149]. To counter this stability challenge, the researchers proposed cryogenic storage at temperatures below −80 °C to prevent destruction of VOCs. They also investigated sample vial coatings using Teflon or glass to reduce VOC adsorption to the internal walls of the vial. These storage and preservation techniques significantly increased stability for cancer-related VOCs in human breath for improved detection [150]. Thus, by applying these storage and preservation methods, they enhanced the stability of cancer-related VOCs in exhaled breath and consequently enhanced the efficiency of disease detection.

### 7.5. Biological Diversity, Matrix Complexity, and the Chemistry of Non-Cancerous VOCs

Importantly, biological variability constitutes another obstacle for the intermediary level as well as for the strategy in general. Metabolic differences, genetic variations, as well as different disease states lead to highly variable VOC profiles, which makes it nearly impossible to recognize specific VOC biomarkers for early cancer diagnoses. Therefore, extensive investigations and rigorous analytical algorithms like machine learning to search for patterns in this variability are demanded to improve VOC-based diagnostics [151].

Another matrix used in VOC detection in cancer diagnostics, particularly in prostate cancer, is urine. Nonetheless, urine is a mixture of many metabolic potential volatile organic and inorganic derivatives (PVOIDs), and its VOC pattern varies in terms of hydration, diet, kidneys, and drug effects [140]. In general, it is very difficult to differentiate VOCs associated with cancer from those corresponding to normal metabolism in the case of prostate cancer detection [59,152]. In a study, researchers used GC-MS to compare urine samples of patients with prostate cancer to control samples. They pointed out several VOCs, such as fatty acid derivatives and aromatic, which were found to be abnormal in prostate cancer patients. However, some of these compounds were also influenced by other parameters besides cancer, such as microbial interference or ingestion of food products. Due to fluctuating VOC levels, certain VOCs that did not provoke evidence of cancer could not be diagnostically linked to the disease [153]. However, due to the complexity of the urine matrices, steps including filtration and controlled storage conditions are other methods employed to reduce matrix interference and degradation of VOCs. The researchers also defined the participants’ diet and medication schedules to minimize variation in VOC profiles they encountered. Further, by expanding the dataset and employing multivariate statistical analyses, the researchers enhanced the ability to diagnose prostate cancer based on urine VOCs [154]. This study demonstrated the need to conduct some pre-processing and sample manipulation to overcome the issues of matrix interference in cancer VOC detection.

### 7.6. Regulatory Hurdles

Furthermore, various validation and regulatory requirements add other challenges, the major of which is the extended time clinicians have to adopt and incorporate into their work routines and normal practice. For VOC detection to be used as one of the normal diagnostic tools in health facilities, easy-to-use, cheap, and portable detection instruments should be designed [155]. Moreover, the corresponding procedures of clinical trials and approvals of such technologies may take substantial time and are frequently rather stringent, which may jeopardize the speed of utilization of the former in the medical field.

## 8. The Development of Modern Electronic Nose Applications for Identifying Cancer VOCs

These artificial olfactory systems are one of the newest technologies resembling the human olfactory system. Such sensor arrays are able to detect VOCs and may be useful in noninvasive diagnosis of various conditions [156]. The potential of this capacity to distinguish not only a singular VOC but also multiple patterns of VOCs is quite promising for cancer detection through the use of fast, effective, and relatively cheap diagnostic devices. The sensor array is indeed the heart of synthetic olfactory systems, which provide a response to the VOCs it comes across. When the sensor surface comes close to VOC molecules, physical or chemical changes happen, which results in the alterations of the properties of the sensor, including conductivity, mass, or frequency [157]. The following information represents how artificial olfactory systems could potentially modify cancer detection by employing sensors.

### 8.1. Metal-Oxide Semiconductor (MOS) Sensors

MOS sensors change their electrical impedance depending on VOCs that adsorb on the surface of metallic oxide. Organic semiconductor surfaces of MOS sensors, such as tin oxide, incorporate oxygen ions. The adsorption of these ions into a VOC molecule affects the concentration of oxygen on the sensor surface, altering the conductivity of the sensor materials. The extent of resistance variation is directly proportional to the concentration of VOCs [158].

### 8.2. Conducting Polymer Sensors

These sensors employ polymers that undergo variation in their electrical characteristics when exposed to VOCs. Molecular structures of conducting polymers such as polyaniline or polypyrrole possess functional groups that react with VOCs by charging transfer or doping to change conductivity. Such interaction varies among VOCs and is based on the chemical properties of VOCs [159].

### 8.3. Quartz Crystal Microbalance (QCM) Devices

QCM sensors measure shifts in mass at the surface of the measuring device. When VOC molecules are adsorbed onto the surface of the sensor, the added mass influences the oscillation frequency of the quartz crystal. This frequency alteration is directly proportional to the concentration of VOC and thus results in a highly sensitive detection method [160].

### 8.4. Surface Acoustic Wave Sensors

VOCs are measured by observing the change in speed at which acoustical waves travel through a piezoelectric material in SAW sensors. As VOCs deposit on the sensor’s surface, they change the mechanical action from the electrical signal across the surface in the form of an acoustic wave. The shift in waves, such as delay or amplitude change, is quantified and established to confirm or reject the presence and concentration of VOC [161].

### 8.5. Carbon Nanotube (CNT)-Based Detectors

CNT sensors are based on the electrical properties of carbon nanotubes as a response to VOCs. When VOCs are adsorbed at the surface of CNTs, the electrical conductance of the nanotubes is altered to offer a proportional signal. Because of its large surface area, these sensors have high sensitivity and can respond quickly [162].

Therefore, artificial olfactory systems are well understood to alter the means through which the diagnosis of cancer is made achievable through the detection of specific VOCs related to malignancies. Tumor cells have specific VOCs resulting from metabolic pathway disruption, leading to a specific “chemical fingerprint” of biological fluids such as breath, urine, or sweat [157]. The e-noses employ an array of chemical sensors which, after the exposure of these VOCs, create electrical signals equivalent to VOC concentration and type. Such signals are analyzed through sophisticated machine learning algorithms and deep learning models that make sensitive and specific discernment of cancer-specific patterns, identifying the type and stage of cancer [163]. This technology has the potential to help detect early cancer, assuming that such a test could be quick, non-invasive, and possibly inexpensive enough to diagnose malignant VOC profiles before cancer-related physical symptoms manifest. The portability of e-noses also offers clinical utility to point-of-care applications and real-time monitoring for high-risk patients. Though complementary to traditional diagnostics, such as imaging and biopsies, e-noses are rapidly moving towards providing full-spectrum multi-cancer screening capabilities within a test [164]. Further, their integrations with artificial intelligence have the potential to optimize the potential for diagnosis, leading to very high throughput and easily accessible and personalized means of cancer detection.

## 9. Conclusions

In order to mitigate cancer-related fatalities, earlier detection and effective therapy are indispensable. Since 90% of cancers possess distinctive advanced indications, it is crucial to investigate innovative techniques for early detection of carcinoma. VOC characterization represents one of the greatest viable techniques where levels of VOC concentration have been shown to be linked to plenty of illnesses, including carcinoma [37]. Discovery of insects detecting external aromas has benefited the identification of sensory receptors, which has also aided in the creation of biological methods for mapping and manipulating sensory networks. Researchers have emphasized the intricate nature of odorant sensilla’s periphery signalling transduction and the astounding amount of biology still to be explored. Nevertheless, the acute sensation, accuracy, and chronological consistency of odorant-inspired neural function are defined not only by sensors (receptors). Undoubtedly, a large number of sensory, non-sensory, and cellular substances involved in this mechanism require to be studied and further characterized [119]. In addition, technological developments are needed to enable intense restriction of several protein molecules in order to differentiate their primary inputs to signalling transduction and their function in sensillar growth. Additionally, various olfactory-linked bio-components, such as OR, ORNs, OBPs, and proteins, have gained demand in the fabrication of olfactory bio-detectors as a result of characteristics of several elements in insect sensory structures being identified throughout research in structural biology, cellular biology, and neuroscience. Among the most promising future techniques for cancer diagnosis is the development of biomimetic sensors inspired by insect olfactory sensilla, which offer exceptional sensitivity, molecular selectivity, and rapid response to VOC signatures. By replicating the microarchitecture, fluidic environment, and neural signaling dynamics of insect olfaction, these platforms have the potential to revolutionize non-invasive diagnostics through real-time, low-cost, and highly specific VOC detection in biological matrices.

## Figures and Tables

**Table 1:** A summary of case reports associated with the analysis of emitted VOCs for the detection of lung cancer.

Cancer Type	Medium	Study Type	VOCs Biomarker	Method/ Technique	Sensitivity	Specificity	Source
Lung Cancer	Exhaled Breath	Clinical Study	3-hydroxy-2-butanone (TG-4)	LC-MS/MS	70%	76%	[41]
			3-hydroxy-2-butanone (TG-4), glycolaldehyde (TG-7), 2-pentanone (TG-8), acrolein (TG-11), nonaldehyde (TG-19), decanal (TG-20), and crotonaldehyde (TG-22)		--	--	
Lung Cancer	Urine	Clinical Study	Nonan-2-one or 5-methylhexan-2-one, heptan-2-one, Benzaldehyde, 5-ethyl-5-methyloxolan-2-one, propan-2-one, 2-methyl-5-methylsulfanylfuran, 3,4-dimethylhexan-2-one, hexane-3,4-dione, Cyclohexanone, Phenol, 4-methylpent-3-enoic acid, 1,2,4-triazole-3,4-diamine, (E)-non-3-en-2-one, (E)-1-(2,6,6-trimethylcyclohexa-1,3-dien-1-yl)but-2-en-1-one, p-Cimene, 5-(3,3-dimethyloxiran-2-yl)-3-methylpent-1- en-3-ol, 2-buta-1,3-dienyl-1,3,5-trimethylbenzene, Pulegone, 2,6,6,10-tetramethyl-1-oxaspiro[4.5]dec-9-ene 3,7-dimethyloctan-3-ol or 3-methylpentan-3-ol, hydroperoxyhexane, 1-(furan-2-yl)ethenone, 2-methoxyphenol, (1,4-dimethylpent-2-enyl)benzene, 1-methyl-4-propan-2-ylcyclohexa-1,4-diene, 1-methyl-4-prop-1-en-2-ylcyclohexa-1,3-diene, 1-methyl-4-prop-1-en-2-ylbenzene or 1-methyl-2-prop-1-en-2-ylbenzene, Carvone, 4,7,7-trimethylbicyclo[4.1.0]hept-2-ene((+)- 4-Carene), 1-methyl-4-propan-2-yl-7- oxabicyclo[2.2.1]heptane, 3,4-dimethylthiophene, 2-(5-ethenyl-5-methyloxolan-2-yl)propan-2-ol, 2,6-dimethyloct-7-en-2-ol, Menthol	HS-SPME-GC-MS	--	--	[42]
Lung Cancer	Exhaled Breath	Clinical Study	Isoprene, Acetone, Dimethylsulfid, 1-Methylthiopropene, Allyl methyl sulfide, 1-Methylthiopropane, 2-Pentanone, Dimethyl disulfide, 2.3-Butandione, Acetonitrile, 2-Butanone, Dimethyl trisulfide, Benzaldehyde, 1-Pentanol, 2-Heptanone, Heptane, Nonanal, Hexane, 3-Heptanone, Octanal, Octane, Toluene, Pentanal, 31 Hexanal, Decane, Dodecane, Undecane, Propylbenzene, Decanal, Heptanal, Butanal Nonane, Benzene, 1.3-pentadiene, Ethylbenzene, 1-Butanol, 1.4-pentadiene, Butyl acetate, o-Xylene, M + p-Xylene	GC-MS	82–88% (ANN Model)	80–86% (ANN Model)	[43]
Lung Cancer	Breath Sample Lung Cancer Tissue Urine	Clinical and Preclinical Studies	--	Statistical software—IBM SPSS version 25.0 (Armonk, NY, USA: IBM Corp)	91.7%	85.1%	[44]
			3(4H)-dibenzofuranone, 4a,9b-dihydro-8,9b-dimethyl-(3(4H)-DBZ)		50.4%	50.1%	
			p-Cresol + 3(4H)-dibenzofuranone, 4a,9b-dihydro-8,9b-dimethyl-(3(4H)-DBZ)		--	--	
Lung Cancer	Exhaled Breath	Clinical Study	Acetaldehyde, Ethanol, Propionaldehyde, Propanol, 2-Hydroxyacetaldehyde, Dimethyl sulfide, Isoprene, Butanal, Benzene, Pentanal, Butyric acid, Toluene, Phenol, Cyclohexanone, Hexanal, Propyl, Styrene, Benzaldehyde, Heptanal;4-hydroxyhexanal, Acetophenone, Propyl cyclohexane, Octanal, Benzothiazole, Nonanal, Decanal, 2,2-Dimethyldecane	HPPI-TOFMS, 18F-FDG PET-CT	82.1%	92.3%	[45]
Lung Cancer	Exhaled Breath	Clinical Study	Butyraldehyde (C_4_H_8_O)	mRMR	SVM 80%	SVM 90%	[46]
					LR 74%	LR 90%	
					kNN 70%	kNN 90%	
					NB 32%	NB 86%	
					DT 58%	DT 78%	
					RF 88%	RF 86%	
					Bagging 82%	Bagging 86%	
					AdaBoost 74%	AdaBoost 76%	
					NN 66%	NN 73%	
			Formaldehyde, Acetaldehyde, Acetone, Isoprenol, Hexanal, 4-Heptanone, Octanal, Hexamethylacetone, Menthol, Undecanal, Dodecyl aldehyde, Tridecanal, Butyric acid, Acetic acid, Cyclopropanone, Ethyl butyrate, Chalcogran, Methylglyoxal, Methyl acrylate, Crotonaldehyde, Methyl propiolate, Cyclopentanone, Benzaldehyde, Cyclohexylmethanone, Geranylacetone, Anthracene-9-carbaldehyde		--	--	

**Table 2:** A summary of case reports associated with the analysis of emitted VOCs for the detection of liver cancer.

Cancer Type	Medium	Study Type	VOCs Biomarker	Method/ Technique	Sensitivity	Specificity	Source
HCC	Exhaled Breath	Clinical Study	Ethanol, Acetone monomer, Dimethyl sulfide, 1,4-pentadiene, Benzene, Isopropyl alcohol, Acetone dimer, Acetonitrile, Toluene		70.0%	88.6%	[47]
			Acetone dimer		95.7%	73.3%	
HCC	Breath Sample	Clinical Study	Phenol 2,2 methylene bis [6-(1,1-dimethyl ethyl)-4-methyl] (MBMBP)	GC-MS	--	--	[48]
HCC	Exhaled Breath	Clinical Study	Acetone; 1,4-pentadiene; methylene chloride; benzene; phenol; allyl methyl sulfide	SPME-GC-MS, SVM	76.5%	82.7%	[49]
			Acetic acid; methyl ester; methylene chloride; phenol; benzene; cyclopentane; pentane		98%	56%	
			Camphene; cyclopentane; methyl; 2-pentanone; dimethyl sulfde; acetonitrile; cyclopentane; 1,3-dimethyl		9.35%	100%	
HCC HCC vs. Fibrosis	Urine	Clinical Study	4-methyl-2,4-bis(p-hydroxyphenyl)pent-1-ene (2TMS derivative); 2-butanone; 2-hexanone; Benzene, 1-ethyl-2-methyl-; 3-Butene- 1,2-diol, 1-(2-furanyl)-; Bicyclo[4.1.0]heptane, 3,7,7-trimethyl-, [1S-(1a,3ß,6a)]-; Sulpiride	GC-IMS, GC-TOF-MS	43%	95%	[50]
HCC vs. Non-Fibrosis					60%	74%	
Fibrosis vs. Non-Fibrosis					29%	90%	

**Table 3:** A summary of case reports associated with the analysis of emitted VOCs for the detection of colorectal cancer.

Cancer Type	Medium	Study Type	VOCs Biomarker	Method/ Technique	Sensitivity	Specificity	Source
CRC	Urine	Clinical Study	Carbon disulphide; Acetone; Ethanol; 2,2,6,6-tetramethyl-4-ethyl-heptane; Dimethyldisulphide; m-xylene; 4-heptanone; Benzenethiol; Pyrrole; 1,6-dichloro-1,5-cyclooctadiene; Biphenyl; Phenol; dibenzofuran	GC-MS	87.8%	88.2%	[51]
				FAIMS	89.9%	77.8%	
				SIFT-MS	77.8%	78.0%	
CRC AAs- Negative Control	Exhaled Breath	Clinical Study	Propyl pyruvate; 2-methylfuran; 2,2,4-tetramethylpentane; p-meth-3-ene; 6-methyl heptane; 2,4-dimethyl pyrrole; Lactic acid; 2-propenoic acid ethenyl ester	GC-MS	79%	70%	[52]
(CRC + AA) − Control					77%	70%	
CRC − Control					80%	70%	
CRC vs. Non-Cancerous (Neural Network)	Urine	Clinical Study	Octanal; Nonanal; Decanal; 2,4-Di-tert-butylphenol; Heptanal; Heptadecane; Undecanal; 3,4-Dimethylcyclohexanol; 5-Hepten-2-ol, 6-methyl-; Hexanal; Acetone; 2-Pentanone; Biphenyl; 2-Heptanone; Cyclopentanone, 2-methyl-; Ethylbenzene; Methane, isocyanato-; Acetophenone; 1-Undecanol; p-Xylene; Benzene; 1-methyl-3-(1-methylethyl)-; Naphthalene; Octane, 2,2,6-trimethyl-		86%	81%	
CRC vs. Non-Cancerous (Random Network)				GC-TOF- MS	89%	75%	[53]
CRC vs. Non-Cancerous (Neural Network)					91%	55%	
CRC vs. Non-Cancerous (Random Network)				PEN3	82%	55%	

**Table 4:** A summary of case reports associated with the analysis of emitted VOCs for the detection of cancer related to digestive system (eosophageal, gastric and pancreatic cancer).

Cancer Type	Medium	Study Type	VOCs Biomarker	Method/ Technique	Sensitivity	Specificity	Source
Esophageal Cancer	Breath	Clinical Study	Indole; Phenol; 1-Propanol; P-cresol; Dimethyl disulfide	SPME-GC-MS	--	--	[54]
Gastric Cancer	Exhaled Breath	Clinical Study	Propanal	MS, PTR- TOF-MS	53.8%	100.0%	[55]
			Aceticamide		61.5%	88.2%	
			Isoprene		84.6%	64.7%	
			1,3-propanediol		73.1%	76.5%	
			Ethylene; Methyl isobutyl ketone; Acetic acid; m-Tolualdehyde; 1,3,5-trimethylbenzene		61.5%	94.1%	
Pancreatic Cancer	Bile	Clinical Study	Bile Samples	FAIMS	100%	77.8%	[56]
Pancreatic Cancer PDAC vs. Healthy	-	-	2-pentanone; Nonanal; 4-ethyl-1,2-dimethyl-Benzene; 2,6-dimethyl-octane; Benzene, 1-ethenyl-2-methyl-	GC-IMS, GC-TOF-MS	72%	96%	[57]
PDAC vs. CP	-	-			38%	88%	
CP vs. Healthy	-	-			38%	96%	

**Table 5:** A summary of case reports associated with the analysis of emitted VOCs for the detection of cancer related to the urinary system (urinary bladder and prostate cancer).

Cancer Type	Medium	Study Type	VOCs Biomarker	Method/ Technique	Sensitivity	Specificity	Source
Urinary Bladder Cancer	Urine	Clinical Study	Butyrolactone, 2-methoxyphenol, 3-methoxy-5-methylphenol, 1-(2,6,6-trimethylcyclohexa-1,3-dien-1-yl)-2-buten 1-one, nootkatone and 1-(2,6,6-trimethyl-1-cyclohexenyl)-2-buten-1-one	SPME, GC × GC-TOF-MS	--	--	[58]
Urinary Bladder Cancer	Urine	Clinical Study	Nonanal; phenol; 5-ethyl-3-methyloxolan-2-one; 2-ethylhexan-1-ol; 1,1,4a-trimethyl4,5,6,7-tetrahydro-3H-naphthalen-2-one; 1-methyl-4-propan-2-ylcyclohexan-1-ol; benzaldehyde; 2,6-dimethyloct-7-en-2-ol	SPME-GC-MS	AUROC (0.77) 71%	AUROC (0.77) 72%	[58]
			Nonanal; 2-ethylhexan-1-ol; 1,1,4a-trimethyl-4,5,6,7-tetrahydro 3H-naphthalen-2-on; 5-ethyl-3-methyloxolan-2-one; 4-methylpent-3-enoic acid; Heptan-2-one		AUROC (0.80) 71%	AUROC (0.80) 80%	
Prostate Cancer	Urine	Clinical and Preclinical Studies	p-Menth-1-en-3-one, 2-Ethyl-1-hexanol, Carvone, 2,4-Di-tert-butyl-phenol, 2,5-Dimethylbenzaldehyde, 4-Heptanone	SPME, GC-MS	75%	69%	[59]

**Table 6:** A summary of case reports associated with analysis of emitted VOCs for the detection of breast and cervical cancer.

Cancer Type	Medium	Study Type	VOCs Biomarker	Method/Technique	Sensitivity	Specificity	Source
Breast Cancer	Urine	Clinical Study	2-nonanone, 4-methil-2-heptanone, Isobutyric acid allyl ester, 1,3-dis-ter-butylbenzene, Benzaldehyde	GC-MS	100.00%	85.71%	[60]
				e-Nose	100.00%	50.00%	
Breast Cancer (MCF-7 (Luminal-A); MCF-10A; MCF-7; MDA-MD-231)	Urine	Preclinical Study	Styrene; oxime-, methoxy-phenyl; benzaldehyde; phenol; aromatic compound; decane; 1-hexanol, 2-ethyl-; benzyl-alcohol; benzeneacetaldehyde; hydrocarbon; decane, 4-methyl-; hydrocarbon; acetophenone; undecane; hydrocarbon; nonanal; dodecane; decanal; benzaldehyde, 3,4-dimethyl; benzene, 1,3-bis(1,1-dimethylethyl)-; decanol; 2-undecanone.	SPME-GC-MS	--	--	[61]
Cervical Cancer	Urine	Clinical Study	4,7,7-Trimethylbicyclo[2.2.1] hepta-2,5-diene; Androst-5-en-3-ol, 4,4-dimethyl-; (3beta)-; Azulene 1,2,3,4,5,6,7,8-octahydro-1,4-dimethyl-7-(1-methylethyl)-; Cyclohexane, 1-ethenyl-1-methyl-2,4-bis(1-methylethenyl); Humulane-1,6-dien-3-ol; Isocyclocitral; Octadecane; Tridecane, 4,8-dimethyl-, between the control group and CC are: 2-Methyl-4-(2,6,6-trimethylcyclohex- 1-enyl)but-2-en-1-ol; 6-Azaestra-1,3,5(10),6,8-pentaen-17-one, 3-methoxy-; Caryophyllene; Cyclopentanol; 3-methyl-2-(2-pentenyl)-, Hexadecane, 1-bromo-; Nonadecane; Thunbergol Neoclovene-(I), dihydro-; (2,6,6-Trimethylcyclohex-1-enyl) acetic acid; 1-Heptatriacotanol; 1-Hydroxy-1,7-dimethyl-4-isopropyl-2,7-cyclodecadiene; 1-Naphthalenepropanol; alphaethenyldecahydro-alpha,5,5,8a-tetramethyl-2-methylene-; 2(1H)-Naphthalenone; octahydro-4a-phenyl-, trans-, 3,5,24-Trimethyltetracontane; Cyclohexanone 3-ethenyl-3-methyl-2-(1-methylethenyl)-6-(1-methylethylidene)-; trans-, Phenol, 4,4′-(1,1-dimethyl-3-methylene-1,3-propanediyl)bis.	GC-MS	91.6%	100%	[62]

## Data Availability

Not applicable.

## Abbreviations

The following abbreviations are used in this manuscript:

18F-FDG PET-CT	18F-fluorodeoxyglucose- positron emission tomography-computed tomography
AAs	advanced adenomas
Amt	ammonium/methylammonium transport
AUROC	area under the receiver operating characteristic
CD36	cluster of differentiation 36
CNT	carbon nanotube
COPD	chronic obstructive pulmonary disease
CP	chronic pancreatitis
CRC	colorectal cancer
cVA	cis-vaccenyl acetate
DMEM	Dulbecco’s modified eagle medium
DT	decision tree
e-nose	electronic nose
FAIMS	high field asymmetric waveform ion mobility spectrometry
GC-IMS	gas chromatography-ion mobility spectrometry
GC-MS	gas chromatography-mass spectrometry
GC-TOF-MS	gas chromatography coupled to time-of-flight mass spectrometry
GIT	gastrointestinal tract
HCC	hepatocellular carcinoma
HPPI-TOFMS	high-pressure photon ionization time-of-flight mass spectrometry
HS-SPME-GC-MS	headspace solid-phase microextraction-gas chromatography-mass spectrometry
IBD	inflammatory bowel disease
IBS	irritable bowel syndrome
IR	ionotropic receptor
IR84a	ionotropic receptor 84a
kNN	k-nearest neighbors
LC-MS/MS	liquid Chromatography-mass spectrometry/mass spectrometry
LFP	local field potential
LIMP2	lysosomal integral membrane protein 2
LR	logistic regression
Mag-HSAE-TD-GC-MS	magnetic headspace adsorptive extraction-thermal desorption-gas chromatography-mass spectrometry
MOS	metal oxide semiconductors
mRMR	maximum relevance minimum redundancy
NB	naive Bayes
NN	neural network
OBP	olfactory binding protein
OR	olfactory receptor
ORCO	olfactory receptor co-receptor
ORN	olfactory receptor neuron
OSN	olfactory sensory neuron
PDAC	pancreatic ductal adenocarcinoma
PEN3	portable electronic nose
PVOIDs	potential volatile organic and inorganic derivatives
PPK25	pickpocket 25
PTR-TOF-MS	proton-transfer-reaction mass spectrometry
QCM	quartz crystal microbalance
RF	random forest
RS	resistant strain
SAW	surface acoustic wave
SIFT-MS	selected ion flow tube mass spectrometry
SNMP1	sensory neuron membrane protein 1
SPME	solid phase microextraction
SVM	support vector machine
TOF-MS	time-of-flight-mass spectrometry
VOCs	volatile organic compounds
